# The potential of functionalized dressing releasing flavonoids facilitates scar-free healing

**DOI:** 10.3389/fmed.2022.978120

**Published:** 2022-10-03

**Authors:** Mengyuan Zhang, Xiaohang Chen, Yuan Zhang, Xiangyu Zhao, Jing Zhao, Xing Wang

**Affiliations:** ^1^School and Hospital of Stomatology, Shanxi Medical University, Taiyuan, China; ^2^Shanxi Province Key Laboratory of Oral Diseases Prevention and New Materials, Taiyuan, China

**Keywords:** flavonoids, scarless regeneration, biomaterials, drug delivery, dressings

## Abstract

Scars are pathological marks left after an injury heals that inflict physical and psychological harm, especially the great threat to development and aesthetics posed by oral and maxillofacial scars. The differential expression of genes such as transforming growth factor-β, local adherent plaque kinase, and yes-related transcriptional regulators at infancy or the oral mucosa is thought to be the reason of scarless regenerative capacity after tissue defects. Currently, tissue engineering products for defect repair frequently overlook the management of postoperative scars, and inhibitors of important genes alone have negative consequences for the organism. Natural flavonoids have hemostatic, anti-inflammatory, antioxidant, and antibacterial properties, which promote wound healing and have anti-scar properties by interfering with the transmission of key signaling pathways involved in scar formation. The combination of flavonoid-rich drug dressings provides a platform for clinical translation of compounds that aid in drug disintegration, prolonged release, and targeted delivery. Therefore, we present a review of the mechanisms and effects of flavonoids in promoting scar-free regeneration and the application of flavonoid-laden dressings.

## Introduction

Scars mark the end of wound healing after tissue defects, and cause physical and psychological harm to individuals depending on the severity and site of the wound (1). When scars appear on the skin, they take away aesthetics; when they appear on the vocal cords, they take away sound; and when they appear postoperatively, they bring about adhesions (2, 3). At the same time, because of the unique position of the maxillofacial area, facial scars can cause functional deficits and, more often than not, aesthetic problems for the patient (4). For example, large burns on the face and neck area frequently result in facial disfigurement, which not only affects the patient’s quality of life but also causes distress to the patient’s mental health (5). The scar therapy economy in the United States will be worth $3.5 billion by 2035, putting a considerable strain on the global health system (6). The most common scar removal methods are laser or hormone treatment, but these treatments are costly and have side effects (7). If scarring could be prevented immediately throughout the wound healing process, patients would suffer less. Researchers have made numerous explorations in this direction in recent years, and a variety of dressings have emerged, loaded with growth factors, stem cells, extracellular vesicles, and other substances to induce tissue regeneration. However, due to uncontrollable potency or dysregulated stem cell differentiation, it is often difficult to move to the clinic, and research often focuses on rapid healing at the expense of controlling post-healing complications (8, 9).

Empirical and anecdotal evidence support the use of herbs in encouraging wound healing, and extensive research into the effect and mechanism of herbs in tissue regeneration is underway (10). However, because of the complicated compound composition, current research tends to focus more on the special natural compounds that are more easily controlled, inexpensive, and biocompatible (11). Hemostasis, inflammatory response, proliferation, re-epithelialization, and remodeling are five processes in the wound healing process that are all regulated by multiple signaling pathways that recruit immune cells, fibroblasts, stem cells, and endothelial cells for repair in response to changes in the regenerative environment (12, 13). These cells work together to trigger signaling pathways such as transforming growth factor-β1 (TGF-β1) that regulate fibroblast proliferation, migration, and collagen synthesis, resulting in fast healing and scarring (14). Flavonoids are a class of molecules that are extensively found in plants and consist of C3-C6-C3 linked carbon chains with two benzene rings, which are classified based on how the two benzene rings are joined, their structure, and the hydroxylation or glycosylation of the benzene rings (15). Flavonoids’ organic bodies have been demonstrated to bind to and regulate the expression of scar-producing genes like TGF-β1, as well as act as antioxidants *via* chemical bonds like phenolic hydroxyl groups, and so have promising anti-scar applications (16). However, a good drug delivery platform that exerts a gradual local release, enhances bioavailability, and improves the physicochemical features of the drug itself, such as water-solubility, is required for clinical translation of flavonoids for scar-free regeneration (17).

Biomaterial drug delivery can improve water-solubility, provide sustained release systems, and enable targeted medication delivery by encapsulating drugs, as well as provide many advantages of their own (18). Ideal biomaterials have antibacterial, anti-inflammatory, and hemostatic properties in addition to providing scaffold and room for cell growth (19). The hydrogel dressing exemplifies the advantages of biomaterials, including absorbing exudate from wounds and providing a moist, sterile environment for wound healing (20). By designing the material as a “sandwich” structure, the exposed part is hydrophobic, which isolates the external stimulus and protects the internal part from tissue contact to release the drug and promote scar-free wound healing (21). Electrostatic spinning technology utilizes electrostatic fields to synthesize nanofiber biomaterials, enhancing the surface area to volume ratio, encouraging cell adhesion and aggregation while reducing bacterial invasion, inflammatory factor expression, scar tissue development, and improving skin tensile strength (22). As novel bio-encapsulation materials, cellulose-based nanoparticles have excellent biocompatibility and facilitate cell-tissue contact with porous architectures to achieve diverse effects such as antibacterial, drug delivery, and wound healing (23). There are also a variety of materials that act as a barrier to aberrant fibroblast adhesion and invasion, allowing for scar-free regeneration (24). However, biomaterials alone lack good efficacy in inducing scarless regeneration on their own, and biomaterials combined with induction factors are a better match (25).

Therefore, we review the current state of flavonoid dressing research, as well as the five dimensions of flavonoids in wound healing: anti-inflammatory antioxidant, antibacterial, antifungal, and regulation of fibroblasts, as well as the current mechanisms of action for flavonoids in scarless regeneration. In addition, we discuss the benefits of biomaterials that release flavonoids, especially some advancements of scar-free regeneration. The mechanism of action of many natural compounds is better understood thanks to the molecular docking experiments and other methods, and the major functional group action features can be gradually generalized. This, combined with the multifunctional biomaterials, will aid in the realization of ideal scar-free regenerative treatments.

## Flavonoids: Sources and health benefits

Flavonoids have the sibling nucleus flavanone (2-phenylchromanone) and are built on a flavonoid backbone of C6 (A ring)-C3 (C ring)-C6 (B ring), where the hydrogen in the backbone is usually replaced by different groups such as hydroxyl, methoxy, and glycosyl groups, which affect their biological activity (26). Depending on the backbone structure and hydroxyl group position, flavonoids, including orangiferin, apigenin, quercetin, catechin, and silymarin, can be classified as flavones, flavonols, flavanones and so on (27). As secondary metabolites of plants, flavonoids are widely distributed in plants; for example, quercetin is abundant in rutin and hawthorn, apigenin in high amounts in chamomile, catechins can be recovered from green tea, and Silybum marianum is produced from the fruits of the plant Silybum marianum. Meanwhile, flavonoids are also widely found in foods such as legumes, fruits, and vegetables, beverages, and especially in herbal medicines. With the development of biotechnology, the purification of flavonoids has become easier and faster (28).

Natural flavonoids have been discovered to exhibit a wide range of biological and pharmacological activities, including antioxidant and anti-inflammatory capabilities, as well as anticoagulant, antiplatelet, anti-obesity, and immunomodulatory properties (29). Furthermore, flavonoids play an important role in the treatment of a variety of diseases, and flavonoid molecules were discovered to inhibit oxidative stress in a study on the molecular mechanisms of neuroprotection, demonstrating good efficacy in the treatment of Alzheimer’s and Parkinson’s diseases (30). Similarly, flavonoids derived from plants (such as buckwheat) have been shown to efficiently lower blood glucose levels and have anti-diabetic properties (31). Flavonoids can also interfere with cell signaling pathways during tumor invasion and growth, lowering cancer risk by preventing tumor cell proliferation and differentiation (32). Furthermore, study of flavonoids, such as epigallocatechin gallate (EGCG), in the field of wound healing have been proved and reviewed comprehensively. For the first time, we’ll look at the progress of using flavonoids to promote scar-free healing in this review.

## Effect of flavonoids to promote scar-free regeneration

**FIGURE 1:**
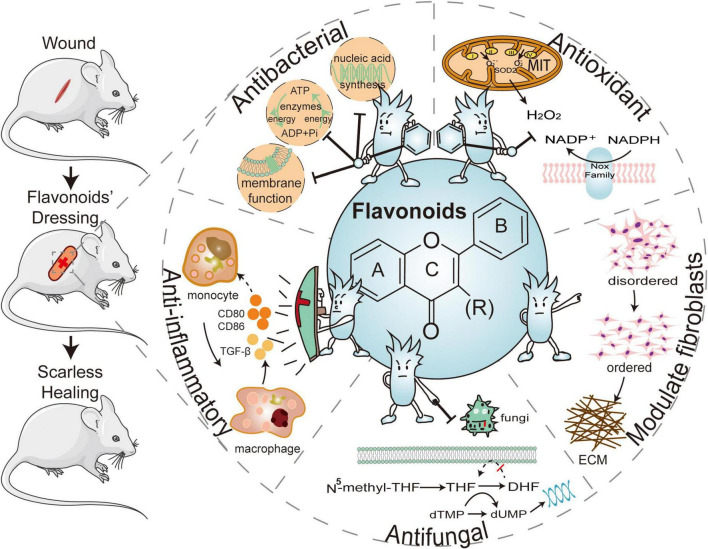
The five major effects of flavonoid-laden dressings are: the hydroxyl substituents of flavonoids can inhibit bacterial nucleic acid synthesis, cell membrane function, and energy metabolism to provide antibacterial functions, as well as the phenolic hydroxyl groups can combine with reactive oxygen species (ROS) to adsorb surrounding free radicals and exert an antioxidant effect. Flavonoids can reduce inflammatory factors, regulating fibroblasts, inhibiting extracellular matrix (ECM) deposition, and promoting scarless healing. Flavonoids can also be antifungal by interfering with folate metabolism and inhibiting biofilm formation. ATP, adenosine triphosphate; ADP, adenosine diphosphate; CD80, cluster of differentiation 80; CD86, cluster of differentiation 86; TGF-β1, transforming growth factor-β1; THF, tetrahydrofolate; DHF, dihydrofolate; dTMP, deoxythymidine phosphate; dUMP, deoxyuridine phosphate; ECM, extracellular matrix; NADPH, nicotinamide adenine dinucleotide phosphate.

Hemostasis, inflammation, proliferation, re-epithelialization, and remodeling are the five basic stages of wound healing, and regulating these processes is expected to achieve scar-free regeneration (33). Bleeding is unavoidable after a skin injury, and then the body triggers a coagulation reaction to stop the bleeding in the first place (34). The blood clot that fills the wound with damaged tissue recruits surrounding immune cells, macrophages, and other cells to phagocyte the “foreigner” and creates a favorable environment for wound healing (35). Neovascularization promotes tissue regeneration and remodeling by delivering nutrients and oxygen to the tissues (36). External factors that negatively influence these processes, such as bacterial infections and oxidative stress, contribute to the formation of scarring (37). Following that, we will discuss the mechanisms by which flavonoids promote wound healing and reduce scarring through anti-inflammatory, antioxidant, antibacterial, antifungal, and fibroblast modulation, respectively (Figure 1).

### Anti-inflammatory

Damaged tissues and clots in wounds will attract inflammatory cells, promoting the absorption and evacuation of necrotic tissue and foreign substances (38). The initial recruited neutrophils trigger the inflammatory response by releasing chemokines, which attract mononuclear macrophages and other innate immune cells to clear cellular debris and microorganisms, and these immune cells eventually leave or undergo apoptosis after the foreign substances are cleared and eliminated (39). Early activation of the inflammatory response aids wound healing, but in order to eliminate the foreign substances quickly, the body responds with an abnormally hyperactive anti-inflammatory response (40). TGF-β1 and platelet-derived growth factor, both released by macrophages and others, activate fibroblasts, and scarring is exacerbated by excessive collagen production or impaired regression (41). For scarless regeneration, regulating inflammation during wound healing is critical (42).

Nuclear factor kappa κB (NF-κB) is a transcription factor that increases the expression of many pro-inflammatory genes in cells, regulates protein kinases, and has a direct impact on cell activation and proliferation (43, 44). The cytokine interleukin-12 (IL-12) promotes the release of the inflammatory factor tumor necrosis factor-α (TNF-α) from immune cells (45). Flavonoids can affect the expression of genes, including NF-κB and IL-12 (46). They also have been proven in numerous clinical trials to considerably reduce TNF-α (47). Furthermore, flavonoids have inhibitory effects on phosphodiesterases, delaying the expression of cyclic AMP (cAMP), a key pro-inflammatory messenger (46).

In addition, flavonoids have long-term effects on immune cell activation and maturation (48). According to studies, macrophages are the key target cells for the anti-inflammatory actions of flavonoids. Whereas, on dendritic cells, flavonoids can block cluster of differentiation 80 (CD80) and CD86, which results in the inhibition of dendritic cell maturation (49, 50). Flavonoids such as apigenin have been shown to have anti-inflammatory properties by decreasing NO release from macrophages and considerably lowering levels of pro-inflammatory cytokines (IL-6 and TNF-α) (51). After quercetin treatment, dense connective tissue formed at the wound of the rat’s injured skin, and the content of C-reactive protein (CRP), an indicator protein for diagnosing inflammatory response, was much lower than in the control group, reducing inflammatory cell infiltration and accelerating wound healing (52). Furthermore, NO production can be used to assess macrophage inflammatory status, and studies have shown that catechins can inhibit NO release in a dose-dependent manner in a limited concentration range (10–40 μg/mL) and exert anti-inflammatory activity on RAW264.7 macrophages (53). Interestingly, when quercetin and catechin were combined, the levels of the two pro-inflammatory cytokines TNF-α and IL-1β were reduced by 78 and 75%, respectively, compared to the control group, significantly higher than when the two were used alone, demonstrating their synergistic anti-inflammatory effects, which were attributed to the inhibitory effect on NF-κB in macrophages (54). Flavonoids may also modulate inflammation selectively, such as EGCG, which is hypothesized to have a pro-inflammatory effect when the level of inflammatory makers is low and a counter-inflammatory effect as inflammatory markers rise (55). These efficacies are better attuned to the various stages of wound healing and help to reduce scarring.

### Antioxidant

During the inflammatory response phase of wound healing, platelets accumulate in large numbers at the wound site and activate neutrophils and macrophages, while the expression of NADPH oxidase in these cells increases dramatically (56). NADPH is produced by neutrophils and macrophages *via* NADPH oxidase, which converts molecular O_2_ in the phagosome to superoxide radical anions, which are rapidly dismutated to hydrogen peroxide (H_2_O_2_), and these chemically active oxygen-containing molecules combine to form reactive oxygen species (ROS) (57). Moderate or basal levels of ROS can maintain normal cellular function and homeostasis and are thought to be crucial for local clearance of foreign bodies by macrophages, but an over-activated inflammatory response can induce excessive levels of ROS (58). ROS have active electrons and are highly reactive in nature, reacting immediately with a wide range of chemicals (59). High levels of ROS near the wound site inhibit cell growth, damage proteins and nucleic acids, cause apoptosis, and prolong the inflammatory process, all of which contribute to scarring (60). Furthermore, high amounts of free radicals cause fibroblasts to convert to the adherent type, which promotes scarring by increasing collagen synthesis and deposition (61). Antioxidant enzymes are able to be activated in a physiological environment, breaking free radicals and reducing ROS, and their increased levels can provide adequate conditions for wound healing. However, in diabetic conditions or in cases of infection, these substances are insufficient to restore the body’s redox state during stress, in which case supplementation with exogenous antioxidants to provide redox homeostasis in cells is essential (62).

Flavonoids have phenolic hydroxyl groups, and the hydrogen atoms on these groups can combine with ROS radicals to generate flavonoid radicals, which attract more radicals to complete the reaction and protect tissue cells from free radical damage (15). The flavonoid catechin has more than 20 times the antioxidant potential of vitamin C (63). Atala et al. evaluated 14 flavonoids and discovered that nine of them could decrease ROS in an alkaline environment by 70% (64). Clinical studies have shown that, after 24 h, patients with nodular disease who received quercetin injections had a 3% increase in total plasma antioxidant capacity, i.e., the total sum of all plasma antioxidants that is expressed as trolox equivalent, and a significant decrease in plasma levels of malondialdehyde, a marker of lipid oxidative damage. These findings suggest that quercetin improves the body’s weakened antioxidant defense system (65). Furthermore, flavonoids inhibit the expression of ROS synthase-related genes or up-regulate the expression of antioxidant synthases (66). Silymarin, a polyphenolic flavonoid lignan, in a random group of patients with type 2 diabetes, and compared to those taking placebo, the oral silymarin group increased superoxide dismutase, glutathione peroxidase activity, and total antioxidant capacity by 12.85, 30.32, and 8.43%, respectively, which greatly improved the *in vivo* antioxidant index and exhibited potential antioxidant properties (67). When compared to controls, patients with pulmonary tuberculosis who received a combination of catechins and antituberculosis showed a significant increase in blood levels of reduced glutathione, an antioxidant effect that plays an important role in the human oxidative stress mechanisms (68). In addition, the levels of ROS metabolites such as F2-isoprostaglandins are considerably reduced when flavonoids are consumed over time (69, 70).

### Antibacterial

Wound infection is a major issue during the healing of skin defects, and the emergence of drug-resistant bacteria poses a challenge for wound debridement and antibacterial agents (71). *Escherichia coli* (*E. coli*) and *Staphylococcus aureus* are common bacteria that cause wound infections, the metabolic substances produced by these bacteria cause inflammation and exudation, for example, the overproduction of matrix metalloproteinases (MMPs) attracts more inflammatory neutrophils into the wound, impeding the growth and migration of cells in the basal skin layer, while a large influx of fibroblasts repairs the void and forms proliferative scars (72). Furthermore, the bacterial metabolite lipopolysaccharide (LPS) is able to significantly reduce local macrophage recruitment to the wound, inhibit wound collagen deposition, and increase apoptotic cells in both the dermis and granulation tissue at the wound edges, resulting in a sustained inflammatory response and prolonged wound healing (73). However, some current antibacterial ingredients may have an impact on the physiological activity of normal cells, for example, silver causes cytotoxicity to keratin-forming cells and fibroblasts subsequently impairing wound healing and leading to scars (74).

Flavonoids’ antibacterial activity is primarily determined by the substituents on the benzene ring, particularly those with hydrophobic substituents, where antibacterial activity is enhanced when the substituent is a hydroxyl group, while methylation of the hydroxyl group reduces antibacterial activity (75). Cushnie et al. reviewed that flavonoids exert antibacterial effects through three mechanisms of action: suppression of bacterial nucleic acid synthesis, cell membrane function, and energy metabolism (76). For example, flavonoids can inhibit pore proteins on the bacterial outer membrane to directly affect the activity of *E. coli*, which is equivalent to cutting off the energy source of *E. coli* such as glucose and amino acids (77). Flavonoids also protect cells by inhibiting bacterial adherence to cells and reducing the production of bacterial toxin products (78). Clinical studies have shown that using flavonoid-rich mouthwash reduces the oral plaque index and the number of *Streptococcus mutans* bacteria, effectively inhibiting the formation of dental plaque biofilm (79). Acne patients used quercetin patches versus placebo preparations on the right and left sides of their faces, respectively, and after 8 weeks, the quercetin group had a 14.7 and 52.9% reduction in the number of acne and total lesions, indicating that quercetin has good antibacterial activity against *Propionibacterium acnes* while being completely safe for fibroblasts (80). Meanwhile, in animal models, Vikram et al. treated rats infected with *Salmonella typhi* with the flavonoid naringenin and found that naringenin specifically inhibited 24 genes in the pathogenicity island of *Salmonella* and attenuated the virulence and cell motility viability of the bacteria (81). In addition to inhibiting bacterial activity through direct inhibition, flavonoids have been shown to exert synergistic effects with antibiotics, such as inhibiting the expression of β-lactamases that produce antibiotic resistance in bacteria (82). To inhibit drug resistant bacteria, flavonoids can serve as inhibitors of bacterial efflux pumps and virulence factors, for example, quercetin strongly reduces extracellular matrix targeting to disrupt *E. coli* colonic biofilms, whereas apigenin restores antibiotic susceptibility to drug-resistant bacteria and limits the spread of drug-resistant bacteria by activating the host innate immune system (83). Also, flavonoids have antibacterial effects in slightly alkaline conditions. The phenolic hydroxyl groups of EGCG are deprotonated and microorganisms are scavenged by redox processes in buffer solutions with pH values near to or greater than the pKa of EGCG (pH 7.4 and 8.0) (84).

### Antifungal

Fungi can quickly invade burns and severely traumatized skin wounds, resulting in widespread wound infections (85). Fungal cell walls are made up of many layers of carbohydrates such α-mannan and β-glucan (86). α-Mannan binds to dendritic cell-associated C-type lectin-2 (dectin-2), activates the NF-κB pathway, induces overproduction of the inflammatory cytokine TNF-α, and inhibits angiogenesis and myofibroblast proliferation (87). Furthermore, the fungus’s mycelium and its elastic biofilm can impede wound healing (88).

Natural flavonoids of plant origin can cause apoptosis and reduce biofilm formation, resulting in antifungal actions *via* multi-targeting (89). Flavonoids destroy fungal biofilms by blocking the essential enzyme isocitrate lyase (ICL), which permits *Candida albicans* to exist and proliferate in a nutrient-limited environment within phagocytes such as macrophages and neutrophils, resulting in cell shrinkage and internal component leakage (90). Meanwhile, flavonoids can inhibit the folic acid synthesis pathway and prevent fungal reproduction. Navarro-Martinez et al. co-cultured EGCG with dihydrofolate reductase from *Candida albicans* at various concentrations and found an inhibition constant (Ki) of 0.7 M, indicating that EGCG can inhibit fungal cell membrane ergosterol production by interfering with folic acid metabolism and with azole antifungal effects in synergy with azoles (91). Furthermore, clinical trials utilizing a rutin-rich plant ointment to treat canine wounds revealed much higher wound retraction rates than controls, as well as powerful antifungal activity against *Candida krusei* around the wounds (92).

### Modulation of fibroblasts

The amount of collagen accumulated was proportional to the number of mobilized fibroblasts (93). The proportion of En1-positive and negative fibroblasts in skin defects is the main cause of scarring (94). En1-positive fibroblasts are activated by inflammation, ROS, bacteria, and wound tension (58). Furthermore, recruitment of deeper dermal fibroblasts, which are larger, slower to proliferate, and secrete vast amounts of collagen fibers while inhibiting collagen degradation through reduced release of cellulase, occurs when wounds are deeper and under greater tension (95). The activation of these pro-scar-forming fibroblasts is a mechanism for the organism to protect against infection and recover quickly, yet scars can become a repair endpoint due to the large synthesis and deposition of extracellular matrix (96).

In addition to regulating inflammation, ROS, and infection during wound healing, flavonoids are also engaged in how to enhance healing without producing excessive fibrosis and scar formation. Flavonoids have been proven in studies to lower TGF-β1 and IL-1β production, limit extracellular matrix (ECM) secretion, and prevent excessive fibrous connective tissue deposition (97). For example, while maintaining the viability of fibroblasts, safranin inhibits TNF-α expression, reduces ECM protein synthesis and improves wound healing (98). Pinocembrin, the most abundant flavonoid compound in propolis, has anti-fibrotic properties, inhibiting TGF-β1 signaling and impairing TGF-β1-induced proliferation and activation of abnormal skin fibroblasts, effectively alleviating bleomycin (an antibiotic)-induced excessive skin fibrosis (99). Furthermore, quercetin treatment of mice wounds resulted in the same duration and rate of wound healing as the control group, but with less ECM deposition *in vivo*, and quercetin was able to modify effective adhesion and migration of fibroblasts as well as resist scar tissue fibrosis (100).

## Flavonoids’ mechanism for scar-free regeneration

Transforming growth factor-β (TGF-β), which comes in three types: TGF-β1, 2, and 3, is the most essential regulatory factor in tissue repair. TGF-β1 is highly expressed in adult wound healing, but TGF-β3 is broadly expressed in newborn and child wound healing (101). TGF-β1 and TGF-β3 share receptors but have opposing roles in scar repair (102). TGF-β1 stimulates fibroblast activation, proliferation, and anti-apoptosis by phosphorylating smad proteins and facilitating cell aggregation at the wound site (103). TGF-β1 activation also boosts fibroblast collagen fiber synthesis while lowering collagenase activity, and TGF-β1 stimulates fibroblasts to develop into myofibroblasts, which compress the wound and induce scar formation (104). Specific TGF-β1 inhibitors are currently being developed, but TGF-β1 serves a crucial physiological function in cells, direct blockage will result in aberrant cell behavior such as apoptosis (105). Natural compounds have many groups on them, and different groups play different roles, forming hydrogen bones, π bonds, or other forces with various genes to enhance or inhibit their expression. The flavonoid icariin has been demonstrated to block the development of the smad-2/3/4 complex and down-regulate the production of calmodulin *via* lowering TGF-β1 activity (106). The capacity of catechins to selectively regulate TGF-β1 and TGF-β3 expression is promising, but the exact mechanism must be explored in its organic form (107).

Transforming growth factor-β1 (TGF-β1) can also activate phosphatidylinositol 3-kinase (PI3K), which causes activation of protein kinase B (Akt), and an abnormally active Akt will promote proliferation and motility of fibroblasts and speed up the formation of proliferative scarring (103). Naringin has been demonstrated to minimize proliferative scarring by decreasing the PI3K/Akt signaling pathway followed by inhibiting fibroblast proliferation and motility (108). Mast cells activate PI3K/Akt/mTOR, which stimulates fibroblast type I collagen expression, whereas EGCG suppresses this process (109). Signal transducer and activator of transcription 3 (STAT3) is important for fibroblast proliferation, migration, and collagen synthesis (110). EGCG was discovered to influence fibroblast proliferation and migration primarily through its inhibitory effect on STAT3, which causes reversible cell cycle when inhibited by PI3K and STAT3 inhibitors (111).

Activin receptor-like kinase 5 (Alk5) is also involved in scar formation, and Alk5 knockdown in a wound healing model resulted in a considerable reduction in scars (112). When TGF-β1 is active, Alk5 is activated in a cascade, and transcription of pro-fibrosis genes begins (14). At lower doses (5.7°μM), baicalein can efficiently inhibit Alk5, although not all flavonoids have this inhibitory activity, which is related to their structure (113). For example, flavonoids with a 5, 7, 3′, 4′ hydroxyl substitution, such as lignan, quercetin, and yohimbine, have a strong affinity for Alk5 and can effectively block collagen expression (114). In addition, luteolin also inhibits Alk5 expression and reduces fibroblast activation for suppressing the TGF-β1/smad pathway in a dose-dependent manner (114).

**FIGURE 2:**
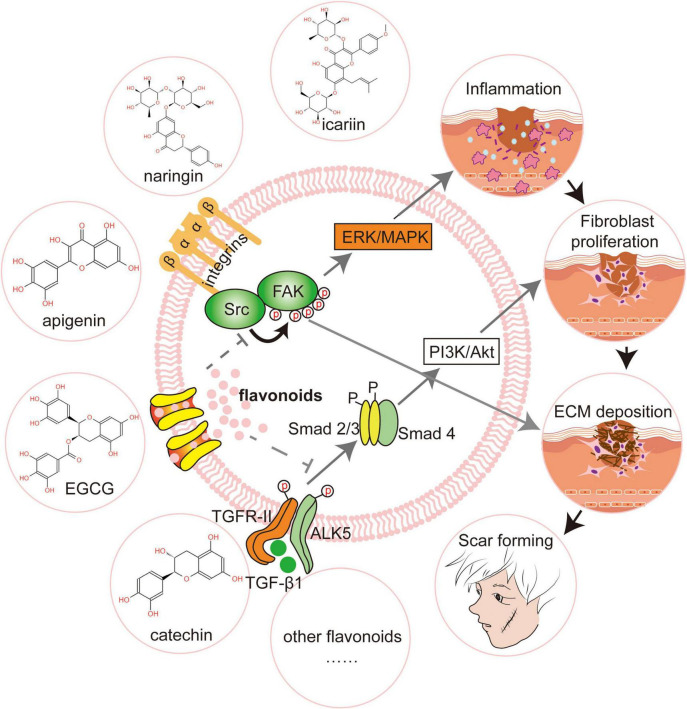
Flavonoids promote scar-free regeneration by regulating signaling pathways. Flavonoids like icariin and epigallocatechin gallate (EGCG) limit fibroblast proliferation and motility by modifying transforming growth factor-1 (TGF-1) and so suppressing the PI3K/AKT signaling pathway. Apigenin drastically lowers focal adhesion kinase (FAK) activity and, as a result, changes the phosphorylation of extracellular signal-regulated kinase (Erk1/2). Catechin, by binding to Scr, reduces extracellular matrix deposition and enhances scar-free wound healing. FAK, Focal Adhesion Kinase; Src, Sarcoma gene; Erk, Extracellular regulatxory protein kinase; MAPK, Mitogen-activated Protein Kinase; Alk5, Activin receptor-like kinase 5; PI3K, Phosphatidylinositol 3-kinase; Akt, Protein kinase B.

Integrins αV and β1 control fibroblast proliferation, migration, and the production of extracellular matrix (115). Focal Adhesion Kinase (FAK) is an essential step in the integrin-mediated signaling cascade during integrin-extracellular matrix interactions, and it promotes wound healing to form scars (116). FAK knockout mice had much lower levels of fibrosis and inflammation, as well as less scarring. FAK inhibition has emerged as a unique strategy for preventing scarring (117). Quercetin and other antioxidants have been demonstrated to increase integrin αV expression while lowering integrin β1 in dermal cells by phosphorylating two sites, 397 and 861, of the FAK complex (118). Apigenin inhibited FAK activity and phosphorylation in human fibroblasts by regulating integrin protein levels, which then affected phosphorylation of extracellular regulatory protein kinase1/2 (Erk1/2), which has important implications for fibroblast proliferation and survival (119). Furthermore, the sarcoma gene (Src) is a complex kinase that frequently works in a functional protein complex with FAK. The placement of Src at the wound’s edge is linked to the early inflammatory response and late regenerative remodeling (120). Src inhibition resulted in decreased myofibroblast and macrophage aggregation, as well as a significant reduction in extracellular matrix deposition and scarring (35). Molecular docking investigations revealed that flavonoids like quercetin, apigenin, and catechin have a high binding energy to Src (121; Figure 2).

## Bioencapsulation overcome the dilemma of flavonoids alone

When flavonoids are utilized alone to regenerate tissue, they frequently encounter issues such as limited water solubility, low bioavailability, and unstable physicochemical qualities. Encapsulating chemicals with biomaterials can improve their water solubility and stability, make them easier to absorb in the body, avoid premature degradation, and prolong circulation time. Furthermore, after being bioencapsulated, these chemicals will effectively target recipient cells, improve their penetration in damaged tissues, and increase therapeutic bioavailability, lowering toxicity (122). Collagen, elastin, hydrogel, and other biomaterials, when loaded with flavonoids, will help provide scaffolds to encourage the orderly proliferation and migration of cells, in addition to the drug’s benefits (123). Therefore, the use of flavonoids and other chemicals in combination with biomaterials is predicted to improve drug water solubility, increase drug loading, provide a sustained-release platform, and enable targeted administration.

### Promoting drug stability and water solubility

Flavonoids’ water solubility is determined by the existence or lack of glycosidic linkages, and their bioavailability is considerably decreased due to the presence of pH, enzymes, and other nutrients in the microenvironment, which often destabilize them (124). Quercetin and rutin, two frequently used flavonoids, have limited water solubility and difficulties penetrating the lipid bilayer of cell membranes, limiting their bioavailability despite their anti-inflammatory and anti-oxidative stress properties in wound healing. Cyclodextrins contain hydrophobic interior and hydrophilic external structures, and with the help of water-soluble external surfaces, they can form highly soluble inclusion complexes. By freeze-drying and solvent evaporation, Başaran et al. prepared inclusion complexes of quercetin and rutin with hydroxypropyl—cyclodextrin, and *in vitro* tests revealed that the concentrations of both in aqueous solution increased from 1.5 and 34.3 μg/mL to 945.6 and 1901.4 μg/mL, respectively (125). Polyphenols (such as EGCG, curcumin, and resveratrol) are stable in acidic environments but degrade in neutral or slightly alkaline environments, whereas nanoparticles can encapsulate bioactive compounds to improve permeability of cell membranes. And when nanoparticles are used to encapsulate polyphenols, their pH stability, water solubility, bioavailability, anti-inflammatory, and antioxidant bioactivities are all improved, and drug degradation is prevented (126). For example, poor water solubility, low absorption, and quick enzymatic degradation limit curcumin, a polyphenolic molecule, *in vivo* (127). Solid lipid nanoparticles with good biocompatibility and stability mixed with polyethylene glycol (PEG)-based emulsifiers can be a carrier for curcumin powder and promote rapid curcumin penetration into the epithelium and increase the aqueous solubility of curcumin powder from 14 to 92–95% (128).

### Drug slow release platform

Topical application of pure compounds has a short duration of action, low stability, and can cause undesirable side effects, whereas sustained release of bioactive drugs can maintain drug levels *in vivo* for a long time with little changes (129). The drug concentration can be kept within the therapeutic range by encapsulating the drug in carriers such as particles, nanoparticles, and hydrogels to release the drug at a steady rate, extending the duration of action and enhancing the drug’s bioavailability (130). Chitosan (CS) is a natural, biodegradable, and biocompatible macromolecular molecule. *In vitro* release results of chitosan nanoparticles (CS-NPs) prepared by the ionic gelation method as an effective carrier for quercetin revealed that the release of quercetin was 29.68% within the first hour, which is significantly lower than that of its application alone, and that the release profile leveled off after 2 h. Furthermore, in the tumor microenvironment (pH = 5.3 and 40°C), the late release rate of quercetin is much higher, with a cumulative drug release percentage of 75.64% within 12 h. The ionic interaction between quercetin and CS may be responsible for the prolonged release feature (131). Meanwhile, Bose et al. produced quercetin nanostructured lipid carriers using solvent (chloroform/acetone) emulsification technology, allowing quercetin to be released biphasically from physiological liposomes up to 24 h. The pace of discharge comes to a halt between 24 and 30 h. This technique boosted skin tissue repair by prolonging the effect of quercetin in wounds (132). Due to its instability and pH sensitivity, EGCG, a physiologically active tea polyphenol in green tea, has a high rate of breakdown and limited bioavailability *in vivo*. Maize protein can encapsulate lipophilic compounds and capture a large number of hydrophobic compounds, and its low gastrointestinal absorption rate can improve the carrier’s controlled release capabilities. The cumulative release of EGCG in simulated gastric and simulated intestinal fluids was 19 and 92%, respectively, after combining with amphiphilic molecular lecithin to prepare maize protein-lecithin-EGCG composite nanoparticles, and increased slowly with time, demonstrating a stable slow release performance (133).

### Targeted drug delivery

To maximize drug bioavailability, it is necessary to facilitate targeted drug administration. Encapsulating the drug in biomaterials and surface functionalization, such as aptamer modification, are two methods for achieving targeted drug delivery. Furthermore, depending on the parameters of the milieu, such as the pH of the microenvironment, or with the addition of external auxiliary light and heat, bioactive substances can be supplied to the target region to boost therapeutic effects and prevent adverse effects (134). Due to rapid metabolism, quercetin, as one of the most prevalent flavonoids, has low targeting efficacy. It does not easily concentrate intracellularly and is easily and quickly eliminated by the organism. The synthesis of phenylboronic acid conjugated zinc oxide nanoparticles (PBA-ZnO) using 3-carboxyphenylboronic acid (PBA) as an aptamer for targeting tumors would serve as an effective carrier for quercetin, while ZnO nanoparticles tend to accumulate in the acidic tumor microenvironment but have limited penetration. The slightly alkaline conditions retain the hydroxyl groups of quercetin ionized, increase tumor cell death, and inhibit the proliferation of murine breast cancer cells, so this technique can successfully target quercetin delivery to sialic acid overexpressing cancer cells (135).

CD44 has a significant affinity for hyaluronic acid (HA) and is overexpressed on the surface of different tumor cells. Mu et al. created HA-EGCG as an adriamycin delivery vehicle by incorporating EGCG into a CD44-modified HA backbone with disulfide links. Targeting EGCG to tumor locations boosts the tumor treatment effect of adriamycin and magnifies the effect of oxidative stress (136). Fucoidan is found in a variety of marine species, most often isolated from brown algae, and exhibits anti-inflammatory, antioxidant, and anti-cancer properties. It can be utilized as a ligand to bind to the scavenging receptor on macrophages’ surfaces, and it can be polymerized with HA to target the CD44 receptor on macrophages’ surfaces, which inspires HA/fucose complexes with PEG-gelatin enclose EGCG. EGCG can be successfully administered to macrophages *via* the dual-targeted binding method of CS and HA to macrophages. Immunofluorescence tests revealed that following encapsulation, EGCG concentration in macrophages increased dramatically, and intensity increased in a time-dependent way, improving EGCG’s on-target delivery capabilities (137).

Furthermore, photothermal treatment has a higher tissue penetration rate and is used in active drug delivery systems. By combining the tumor-targeting molecule folate (FA) with silver nanoparticles (AgNPs) loaded with quercetin (QRC), Bose et al. prepared folate receptor-targeted silver nanoparticles (QRC-FA-AgNPs) and the fluorescence intensity of target cells cultured with QRC-FA-AgNPs was much higher than cells cultured with AgNPs alone, effectively promoting targeted drug delivery and achieving combined therapeutic effects, providing a new idea for targeted drug therapy (138).

### Tissue regeneration scaffolds

The repair of tissue defects caused by trauma, infection, and surgery is dependent on a number of processes that are disrupted by the organism’s internal and external environment, whereas biomaterials such as hydrogels, collagen, extracellular matrix, and other biomaterials will direct the arrangement and growth of cells due to their ordered porous structure. As such, in combination with induction actions such as drugs, their extra anti-inflammatory and antibacterial properties should result in a beneficial wound healing impact (139). The average cell viability of human dermal fibroblasts is 76% by electrostatic spinning to develop polymeric nanofiber scaffolds with a multi-microporous structure, which is much higher than the level of the control group without nanofibers, and could be used effectively for wound healing with reduced cytotoxicity (140). Chitosan has high biocompatibility and promotes cell attachment and proliferation. Vedakumari et al. prepared chitosan-fibronectin composite scaffolds loaded with quercetin, and found that a large number of fibroblasts and epithelial cells migrated to the wound site, that fibroblasts proliferated faster, that the time required for complete wound epithelialization decreased from 29 to 16 days, and that the scaffolds had good bactericidal activity against *E. coli* and *Staphylococcus* (141).

While adjuvant decellularized dermal matrix (ADM) collagen scaffolds can serve as effective carriers for quercetin and their high porosity can increase the contact area between cells and the scaffold surface and accelerate mesenchymal differentiation, functionalization of graphene oxide (GO) with PEG to synthesize GO-PEG nanocarriers can improve drug delivery efficiency but poor cell induction. The ADM collagen scaffold’s high porosity can improve the contact area between cells and the scaffold surface, speed up MSC adherence and proliferation, and boost collagen deposition and angiogenesis in wound healing (142). Croitoru et al. used electrostatic spinning to create a quercetin fiber scaffold matrix based on polylactic acid (PLA) and GO, and electron microscopic observations revealed that cultured L929 fibroblasts increased in density, adhered uniformly to the scaffold in a circular shape, and reached a maximum survival rate of 82.3% within 7 days. Additionally, quercetin in the PLA/GO scaffold matrix can stimulate IL-6 production in fibroblasts, modulating the acute cellular inflammatory response and accelerating wound healing (143).

## Flavonoid-laden dressings for scar-free regeneration

Many of the ways outlined above demonstrate the significant benefits of biomaterials for drug administration, which can increase drug bioavailability and stability while also promoting tissue regeneration and wound healing. By obtaining a large number of polymers and bioactive compounds from natural resources such as plants, modern wound dressings have developed various types of antibacterial dressings, such as hydrogels, films, scaffolds, fibers, sponges, and other biomaterials, which provide excellent wound healing effects (144). Biomaterials can also mimic the extracellular matrix and influence cell behavior such as migration and proliferation, resulting in a moist, sterile wound healing environment that promotes tissue regeneration synergistically (145). Many studies are currently being conducted to determine the effect of flavonoid-rich dressings on scar-free regeneration.

In chronic deep second degree burns infected with *Pseudomonas aeruginosa*, catechin-loaded nanocollagen dressings exhibited antibacterial and pro-angiogenic properties, and regeneration of skin appendages and orderly collagen tissue alignment were seen, which may be the result of selective modulation of TGF-β1 and TGF-β3 by catechins (146). One study combined flavonoids into lipid nanoemulsions, which increased the viability of keratin-forming cells and their ability to migrate to wounds, accelerating scar-free skin regeneration (147). In a rat wound model, adding isoflavone glycosides to the nanoemulsion increased keratinocyte viability up to concentrations of 0.5 μg/mL. TNF-α content was also decreased, which reduced the inflammatory response while promoting re-epithelialization and angiogenesis in skin tissue (148). Furthermore, after treating human skin wounds with quercetin-encapsulated nanoemulsions and hydrogels, mature collagen fibers were regularly oriented in parallel and well-organized reticular dermis, and the wound surface produced an intact epithelial layer with covering scars (149). Jin et al. created quercetin-modified silicone gel sheets and tested them on a rabbit ear skin wound model, finding lower expression of type I and type III collagen as well as more effective inhibition of fibroblast proliferation in scar tissue (150). Wu et al. prepared soluble microneedles using cyclodextrin metal-organic scaffolds loaded with quercetin and encapsulated with fibroblast membranes, which were dispersed in HA polysaccharide, a modification with the ability to target fibroblasts, providing a new strategy for drug delivery systems in proliferative scars (151).

Surgical adhesions are scar that form within tissues as a result of surgical procedures, infections, and other factors, leading to severe organ malfunction and chronic discomfort, as well as a significant financial burden on the healthcare system (152). Dressings containing flavonoids have demonstrated good effects in terms of inflammatory, infectious, and anti-fibrotic effects on the adhesion development process. Shin et al. used a poly(lactide-co-glycolide) (PLGA) electrospun scaffold loaded with EGCG, and the dressing showed equivalent anti-adhesive effects to commercial tissue adhesion barriers while being cost effective (153). Lee et al. also found that the EGCG-loaded PLGA barrier film inhibited fibroblast growth and adhesion as well as macrophage release of pro-inflammatory cytokines, resulting in good anti-abdominal adhesion effects (154). After lumbar laminectomy, Huang et al. created an electrostatically spun membrane with polyhexolactone and collagen, into which icariin was loaded as an anti-adhesion barrier membrane. Within 1 week, the barrier membrane released icariinin response to the large proliferation of fibroblasts during the inflammatory phase, and the expression of TGF-β, smad2/3, and collagen fiber deposition were considerably reduced. The opening in the unloaded icariin grouped barrier membranes was filled with a substantial amount of fibrous tissue, but the loaded icariin barrier membranes recovered effectively and had an excellent anti-adhesive action. The electrostatic spinning membrane not only organized fibroblast penetration and adhesion, but it also offered a good platform for the flavonoid icariin to limit drug release and reduce fibroblast growth and collagen deposition (155).

## Challenge and outlook

Skin injuries happen every day, and the scars that remain after the lesion heals cause regret in both cosmetic and physiological aspects. There is a plethora of research on the speed of skin injury recovery, much of which focuses on the treatment of chronic infected wounds, with scar-free regeneration receiving less attention. As everyone’s quality of life improves, we must not just focus on wound healing speed but also on leaving patients with the fewest regrets possible—including striving for scar-free healing. While numerous multifunctional dressings have demonstrated outstanding results in anti-inflammatory, antibacterial, antioxidant, and angiogenic functions to date, fibroblast regulation and antifibrosis have received less attention. Scarogenesis’ mechanisms have steadily been clarified in recent years, and it is currently thought that the phenotypic alteration of fibroblasts is the “culprit” in the scarogenesis process. Future dressings will be more targeted as the mechanics of scarogenesis are better understood and the genes that regulate scarogenesis are revealed.

### Exploration of optimal anti-scarring moiety

Natural substances provide a plethora of possibilities for scar-free regeneration. Due to their phenolic hydroxyl groups, flavonoids are believed to be a strategy for scar-free regeneration through antioxidant, antibacterial, anti-inflammatory, and anti-fibrotic activities during wound healing. However, it’s vital to be aware that flavonoids come in a wide variety of forms, and not every flavonoid has the effects of scarless regeneration. Molecular docking can correctly predict the effective conformational areas and locations of action of drugs (156). Finding the primary sites of action of compounds that have the same effect can be helpful in the creation of novel medications. The exploration and discovery of the “optimal anti-scarring moiety” is highly expectation because it implies (1) the ability to avoid the time-consuming extraction process and synthesize directly in the laboratory for better cost and batch control; (2) the ability to directly incorporate the moiety into biological materials through chemical grafting, for integrated encapsulation and maximum bioavailability; (3) avoiding further harmful groups’ detrimental impacts; (4) increasing the solubility of medicines in water; and so on.

### Extraction and separation of flavonoids

The extraction and separation of certain chemicals from flavonoids is also a significant barrier to their utilization. Flavonoids are mostly found in plants as glycosides and free sapogenins, and crude extracts are obtained by using organic solvents like methanol and concentrated by rotary evaporator, followed by separation and characterization of pure bioactive compounds by liquid chromatography and spectroscopy, for example, quercetin from ethyl acetate extract (157). However, raising the temperature during the extraction process can increase the water solubility of alcohol contaminants and interfere with the rate of flavonoid leaching. Moreover, high temperatures may promote oxidation and breakdown of the structure of flavonoids, resulting in a decrease in their extraction rate. How to decrease the impact of temperature and other external influences while efficiently isolating quercetin, catechin, and other pure components have become a pressing issue for us to explore. In addition to the extraction of flavonoids, it is critical to distinguish between the hydrophilic and hydrophobic qualities of certain flavonoids. For example, the flavonol quercetin is hydrophobic, whereas the flavane-like catechin is water soluble. Clarifying flavonoid water solubility qualities is essential for investigating the corresponding hydrophilic or hydrophobic encapsulation carriers, crosslinking synergistically to boost drug release, and improving flavonoid bioavailability.

### Combination of flavonoids biomaterials

Furthermore, the subject of biomaterials is quickly evolving and confronts numerous obstacles, such as mass production, biosafety, and biodegradability. Hydrogels and extracellular matrix, for example, can all play a scaffolding function in wound healing and can also help with drug dissolution and targeted distribution. However, it is debatable whether the potential cytotoxicity of biomaterials and the possibility of biomaterials interfering with the anti-scarring effect of flavonoids. The application of some ingredients may play a negative role in promoting scar formation, such as silver, with its good antibacterial effect and remarkable efficacy in promoting wound healing, but its potential toxicity to cells can exacerbate scar formation. As such, more research is also needed to determine the optimum flavonoid-biomaterial combinations.

Fortunately, several clinical investigations on the use of flavonoids for scar-free regeneration are currently underway. In comparison to direct injection of epidermal growth factor, EGCG dramatically decreased human scars *in vitro*, exhibiting a good trend of improving scar thickness and skin flexibility (158). Commercial use has also been granted to certain flavonoids-containing gels, such as cumene glycosides. As research develops, the biological properties of flavonoids will be further clarified, and their combination application with biomaterials will become one of the ideal alternatives for scar-free regeneration of skin wounds.

## Conclusion

Flavonoids are abundant and widely available in nature, and their anti-inflammatory, antibacterial, and antioxidant properties contribute to their antitumor and antidiabetic medicinal value. The antioxidant effect of phenolic hydroxyl groups formed by the benzene ring and the hydroxyl groups in flavonoids has been shown to be important in promoting wound healing, but little attention has been paid to the role of flavonoids in modulating scar production. In this review we systematically summarize the effects and mechanisms by which flavonoids exert scar-free effects. Flavonoids may regulate FAK, TGF-β, integrins, and Alk (which are all important genes for scar production) mainly by affecting fibroblasts in wound healing, and these processes reduce abnormal deposition of collagen fibers during wound healing and subsequently promote scar-free healing.

On the other hand, flavonoids applied to local wounds alone, have low local bioavailability, poor water solubility, and are unable to trigger ordered tissue regeneration. The use of biomaterials to encapsulate flavonoids gives us new ideas to help tackle these challenges above and offers great potential in the field of scar-free healing. However, researchers will have to think about how to extract more regulation from the structure of this class of chemicals in the future, how to integrate the biomaterial with flavonoids, how to maximize the economic effect. More importantly, since the scar formation cycle in animals differs from that in humans, practical application in humans still necessitates testing for dosage, efficacy, and a variety of other factors. We expect that every initiative we take is a step closer to scar-free regeneration.

## Author contributions

MZ and XC drafted and edited the manuscript. YZ and XZ were involved in the revision of the manuscript. JZ and XW were involved in concept development and editing the manuscript drafts. All authors contributed to the article and approved the submitted version.

## References

[1] Xue M Jackson CJ. Extracellular matrix reorganization during wound healing andits impact on abnormal scarring. *Adv Wound Care (New Rochelle).* (2015) 4:119–36. 10.1089/wound.2013.0485 25785236 PMC4352699

[2] Kumai Y. Pathophysiology of fibrosis in the vocal fold: current research, future treatment strategies, and obstacles to restoring vocal fold pliability. *Int J Mol Sci.* (2019) 20:2551. 10.3390/ijms20102551 31137626 PMC6567075

[3] Fischer A Koopmans T Ramesh P Christ S Strunz M Wannemacher J Post-surgical adhesions are triggered by calcium-dependent membrane bridges between mesothelial surfaces. *Nat Commun.* (2020) 11:3068. 10.1038/s41467-020-16893-3 32555155 PMC7299976

[4] Bennis I Thys S Filali H Brouwere VD Sahibi H Boelaert M Psychosocial impact of scars due to cutaneous leishmaniasis on high school students in Errachidia province. Morocco. *Infect Dis Poverty.* (2017) 6:46. 10.1186/s40249-017-0267-5 28385151 PMC5383955

[5] Chaudhary FA Ahmad B Sinor MZ. The severity of facial burns, dental caries, periodontal disease, and oral hygiene impact oral health-related quality of life of burns victims in Pakistan: a cross-sectional study. *BMC Oral Health.* (2021) 21:570. 10.1186/s12903-021-01923-3 34749722 PMC8573980

[6] Ljubimov AV Saghizadeh M. Progress in corneal wound healing. *Prog Retin Eye Res.* (2015) 49:17–45. 10.1016/j.preteyeres.2015.07.002 26197361 PMC4651844

[7] Ojeh N Bharatha A Gaur U Forde AL. Keloids: current and emerging therapies. *Scars Burn Heal.* (2020) 6:2059513120940499. 10.1177/2059513120940499 32844039 PMC7418256

[8] Zhao J Li X Hu J Chen F Qiao S Sun X Mesenchymal stromal cell-derived exosomes attenuate myocardial ischaemia-reperfusion injury through miR-182-regulated macrophage polarization. *Cardiovasc Res.* (2019) 115:1205–16. 10.1093/cvr/cvz040 30753344 PMC6529919

[9] Chen WC Lee BG Park DW Kim K Chu H Kim K Controlled dual delivery of fibroblast growth factor-2 and interleukin-10 by heparin-based coacervate synergistically enhances ischemic heart repair. *Biomaterials.* (2015) 72:138–51. 10.1016/j.biomaterials.2015.08.050 26370927 PMC4617784

[10] Lordani TVA de Lara CE Ferreira FBP Monich MST da Silva CM Lordani CRF Therapeutic effects of medicinal plants on cutaneous wound healing in humans: a systematic review. *Mediators Inflamm.* (2018) 2018:7354250. 10.1155/2018/7354250 29805312 PMC5901822

[11] Stan D Enciu AM Mateescu AL Ion AC Brezeanu AC Stan D Natural compounds with antimicrobial and antiviral effect and nanocarriers used for their transportation. *Front Pharmacol.* (2021) 12:723233. 10.3389/fphar.2021.723233 34552489 PMC8450524

[12] Morbidelli L Genah S Cialdai F. Effect of microgravity on endothelial cell function, angiogenesis, and vessel remodeling during wound healing. *Front Bioeng Biotechnol.* (2021) 9:720091. 10.3389/fbioe.2021.720091 34631676 PMC8493071

[13] Thapa RK Diep DB Tønnesen HH. Topical antimicrobial peptide formulations for wound healing: current developments and future prospects. *Acta Biomater.* (2020) 103:52–67. 10.1016/j.actbio.2019.12.025 31874224

[14] Frangogiannis N. Transforming growth factor-β in tissue fibrosis. *J Exp Med.* (2020) 217:e20190103. 10.1084/jem.20190103 32997468 PMC7062524

[15] Panche AN Diwan AD Chandra SR. Flavonoids: an overview. *J Nutr Sci.* (2016) 5:e47. 10.1017/jns.2016.41 28620474 PMC5465813

[16] Shan S Zhang Y Wu M Yi B Wang J Li Q. Naringenin attenuates fibroblast activation and inflammatory response in a mechanical stretch-induced hypertrophic scar mouse model. *Mol Med Rep.* (2017) 16:4643–9. 10.3892/mmr.2017.7209 28849050 PMC5647020

[17] Gugleva V Ivanova N Sotirova Y Andonova V. Dermal drug delivery of phytochemicals with phenolic structure *via* lipid-based nanotechnologies. *Pharmaceuticals (Basel).* (2021) 14:837. 10.3390/ph14090837 34577536 PMC8471500

[18] Quazi MZ Park N. Nanohydrogels: advanced polymeric nanomaterials in the era of nanotechnology for robust functionalization and cumulative applications. *Int J Mol Sci.* (2022) 23:1943. 10.3390/ijms23041943 35216058 PMC8875080

[19] Negut I Dorcioman G Grumezescu V. Scaffolds for wound healing applications. *Polymers (Basel).* (2020) 12:2010. 10.3390/polym12092010 32899245 PMC7563417

[20] Op ’t Veld RC Walboomers XF Jansen JA Wagener FADTG. Design considerations for hydrogel wound dressings: strategic and molecular advances. *Tissue Eng Part B Rev.* (2020) 26:230–48. 10.1089/ten.TEB.2019.0281 31928151

[21] Azimi B Maleki H Zavagna L De la Ossa JG Linari S Lazzeri A Bio-based electrospun fibers for wound healing. *J Funct Biomater.* (2020) 11:67. 10.3390/jfb11030067 32971968 PMC7563280

[22] Mulholland EJ. Electrospun biomaterials in the treatment and prevention of scars in skin wound healing. *Front Bioeng Biotechnol.* (2020) 8:481. 10.3389/fbioe.2020.00481 32582653 PMC7283777

[23] Abdelhamid HN Mathew AP. Cellulose-based nanomaterials advance biomedicine: a review. *Int J Mol Sci.* (2022) 23:5404. 10.3390/ijms23105405 35628218 PMC9140895

[24] Keane TJ Horejs CM Stevens MM. Scarring vs. functional healing: matrix-based strategies to regulate tissue repair. *Adv Drug Deliv Rev.* (2018) 129:407–19. 10.1016/j.addr.2018.02.002 29425770 PMC6372066

[25] Chen FM Liu X. Advancing biomaterials of human origin for tissue engineering. *Prog Polym Sci.* (2016) 53:86–168. 10.1016/j.progpolymsci.2015.02.004 27022202 PMC4808059

[26] Chen L Teng H Xie Z Cao H Cheang WS Skalicka-Woniak K Modifications of dietary flavonoids towards improved bioactivity: an update on structure-activity relationship. *Crit Rev Food Sci Nutr.* (2018) 58:513–27. 10.1080/10408398.2016.1196334 27438892

[27] Zhang J Liu Z Luo Y Li X Huang G Chen H The role of flavonoids in the osteogenic differentiation of mesenchymal stem cells. *Front Pharmacol.* (2022) 13:849513. 10.3389/fphar.2022.849513 35462886 PMC9019748

[28] Hughes SD Ketheesan N Haleagrahara N. The therapeutic potential of plant flavonoids on rheumatoid arthritis. *Crit Rev Food Sci Nutr.* (2017) 57:3601–13. 10.1080/10408398.2016.1246413 27874281

[29] Cao H Chen X Jassbi AR Xiao J. Microbial biotransformation of bioactive flavonoids. *Biotechnol Adv.* (2015) 33:214–23. 10.1016/j.biotechadv.2014.10.012 25447420

[30] Küpeli Akkol E Tatlı Çankaya I Şeker Karatoprak G Carpar E Sobarzo-Sánchez E Capasso R. Natural compounds as medical strategies in the prevention and treatment of psychiatric disorders seen in neurological diseases. *Front Pharmacol.* (2021) 12:669638. 10.3389/fphar.2021.669638 34054540 PMC8155682

[31] Yen FS Qin CS Xuan STS Ying PJ Le HY Darmarajan T Hypoglycemic effects of plant flavonoids: a review. *Evid Based Complement Alternat Med.* (2021) 2021:2057333. 10.1155/2021/2057333 34925525 PMC8674047

[32] George VC Dellaire G Rupasinghe HPV. Plant flavonoids in cancer chemoprevention: role in genome stability. *J Nutr Biochem.* (2017) 45:1–14. 10.1016/j.jnutbio.2016.11.007 27951449

[33] Las Heras K Igartua M Santos-Vizcaino E Hernandez RM. Chronic wounds: current status, available strategies and emerging therapeutic solutions. *J Control Release.* (2020) 328:532–50. 10.1016/j.jconrel.2020.09.039 32971198

[34] Carnicer-Lombarte A Chen ST Malliaras GG Barone DG. Foreign body reaction to implanted biomaterials and its impact in nerve neuroprosthetics. *Front Bioeng Biotechnol.* (2021) 9:622524. 10.3389/fbioe.2021.622524 33937212 PMC8081831

[35] Landén NX Li D Ståhle M. Transition from inflammation to proliferation: a critical step during wound healing. *Cell Mol Life Sci.* (2016) 73:3861–85. 10.1007/s00018-016-2268-0 27180275 PMC5021733

[36] Rademakers T Horvath JM van Blitterswijk CA LaPointe VLS. Oxygen and nutrient delivery in tissue engineering: approaches to graft vascularization. *J Tissue Eng Regen Med.* (2019) 13:1815–29. 10.1002/term.2932 31310055 PMC6852121

[37] Cheng H Shi Z Yue K Huang X Xu Y Gao C Sprayable hydrogel dressing accelerates wound healing with combined reactive oxygen species-scavenging and antibacterial abilities. *Acta Biomater.* (2021) 124:219–32. 10.1016/j.actbio.2021.02.002 33556605

[38] Sheikh Z Brooks PJ Barzilay O Fine N Glogauer M. Macrophages, foreign body giant cells and their response to implantable biomaterials. *Materials (Basel).* (2015) 8:5671–701. 10.3390/ma8095269 28793529 PMC5512621

[39] de Oliveira S Rosowski EE Hutten locher A. Neutrophil migration in infection and wound repair: going forward in reverse. *Nat Rev Immunol.* (2016) 16:378–91. 10.1038/nri.2016.49 27231052 PMC5367630

[40] Wang J. Neutrophils in tissue injury and repair. *Cell Tissue Res.* (2018) 371:531–9. 10.1007/s00441-017-2785-7 29383445 PMC5820392

[41] Ueshima E Fujimori M Kodama H Felsen D Chen J Durack JC Macrophage-secreted TGF-β(1) contributes to fibroblast activation and ureteral stricture after ablation injury. *Am J Physiol Renal Physiol.* (2019) 317:F52–64. 10.1152/ajprenal.00260.2018 31017012 PMC6692725

[42] Wilgus TA. Inflammation as an orchestrator of cutaneous scar formation: a review of the literature. *Plast Aesthet Res.* (2020) 7:54. 10.20517/2347-9264.2020.150 33123623 PMC7592345

[43] Mussbacher M Salzmann M Brostjan C Hoesel B Schoergenhofer C Datler H Cell type-specific roles of NF-κB linking inflammation and thrombosis. *Front Immunol.* (2019) 10:85. 10.3389/fimmu.2019.00085 30778349 PMC6369217

[44] Sun SC. The non-canonical NF-κB pathway in immunity and inflammation. *Nat Rev Immunol.* (2017) 17:545–58. 10.1038/nri.2017.52 28580957 PMC5753586

[45] Kany S Vollrath JT Relja B. Cytokines in inflammatory disease. *Int J Mol Sci.* (2019) 20:6008. 10.3390/ijms20236008 31795299 PMC6929211

[46] El-Shitany NA Eid BG. Icariin modulates carrageenan-induced acute inflammation through HO-1/Nrf2 and NF-kB signaling pathways. *Biomed Pharmacother.* (2019) 120:109567. 10.1016/j.biopha.2019 31670031

[47] Ferraz CR Carvalho TT Manchope MF Artero NA Rasquel-Oliveira FS Fattori V Therapeutic potential of flavonoids in pain and inflammation: mechanisms of action, pre-clinical and clinical data, and pharmaceutical development. *Molecules.* (2020) 25:762. 10.3390/molecules25030762 32050623 PMC7037709

[48] Hosseinzade A Sadeghi O Biregani AN Soukhtehzari S Brandt GS Esmaillzadeh A. Immunomodulatory effects of flavonoids: possible induction of T CD4+ regulatory cells through suppression of mTOR pathway signaling activity. *Front Immunol.* (2019) 10:51. 10.3389/fimmu.2019.00051 30766532 PMC6366148

[49] Ginwala R Bhavsar R Chigbu DI Jain P Khan ZK. Potential role of flavonoids in treating chronic inflammatory diseases with a special focus on the anti-inflammatory activity of apigenin. *Antioxidants (Basel).* (2019) 8:35. 10.3390/antiox8020035 30764536 PMC6407021

[50] Liu SH Lin CH Hung SK Chou JH Chi CW Fu SL. Fisetin inhibits lipopolysaccharide-induced macrophage activation and dendritic cell maturation. *J Agric Food Chem.* (2010) 58:10831–9. 10.1021/jf1017093 20923145

[51] Hong S Dia VP Zhong Q. Synergistic anti-inflammatory activity of apigenin and curcumin co-encapsulated in caseins assessed with lipopolysaccharide-stimulated RAW 264.7 macrophages. *Int J Biol Macromol.* (2021) 193:702–12. 10.1016/j.ijbiomac.2021.10.153 34717976

[52] Elloumi W Mahmoudi A Ortiz S Boutefnouchet S Chamkha M Sayadi S. Wound healing potential of quercetin-3-O-rhamnoside and myricetin-3-O-rhamnoside isolated from *Pistacia lentiscus* distilled leaves in rats model. *Biomed Pharmacother.* (2022) 146:112574. 10.1016/j.biopha.2021.112574 35062055

[53] Kuang W Yang J Liu Z Zeng J Xia X Chen X Catechin mediates ferroptosis to exert an anti-inflammatory effect on RAW 264.7 cells. *Foods.* (2022) 11:1572. 10.3390/foods11111572 35681322 PMC9180002

[54] Li T Li F Liu X Liu J Li D. Synergistic anti-inflammatory effects of quercetin and catechin *via* inhibiting activation of TLR4-MyD88-mediated NF-κB and MAPK signaling pathways. *Phytother Res.* (2019) 33:756–67. 10.1002/ptr.6268 30637814

[55] Khalatbary AR Ahmadvand H. Anti-inflammatory effect of the epigallocatechin gallate following spinal cord trauma in rat. *Iran Biomed J.* (2011) 15:31–7. 21725497 PMC3639740

[56] Sanchez MC Lancel S Boulanger E Neviere R. Targeting oxidative stress and mitochondrial dysfunction in the treatment of impaired wound healing: a systematic review. *Antioxidants (Basel).* (2018) 7:98. 10.3390/antiox7080098 30042332 PMC6115926

[57] Schäfer M Werner S. Oxidative stress in normal and impaired wound repair. *Pharmacol Res.* (2008) 58:165–71. 10.1016/j.phrs.2008.06.004 18617006

[58] Dunnill C Patton T Brennan J Barrett J Dryden M Cooke J Reactive oxygen species (ROS) and wound healing: the functional role of ROS and emerging ROS-modulating technologies for augmentation of the healing process. *Int Wound J.* (2017) 14:89–96. 10.1111/iwj.12557 26688157 PMC7950185

[59] Phaniendra A Jestadi DB Periyasamy L. Free radicals: properties, sources, targets, and their implication in various diseases. *Indian J Clin Biochem.* (2015) 30:11–26. 10.1007/s12291-014-0446-0 25646037 PMC4310837

[60] Nita M Grzybowski A. The role of the reactive oxygen species and oxidative stress in the pathomechanism of the age-related ocular diseases and other pathologies of the anterior and posterior eye segments in adults. *Oxid Med Cell Longev.* (2016) 2016:3164734. 10.1155/2016/3164734 26881021 PMC4736974

[61] Piera-Velazquez S Jimenez SA. Oxidative stress induced by reactive oxygen species (ROS) and NADPH oxidase 4 (NOX4) in the pathogenesis of the fibrotic process in systemic sclerosis: a promising therapeutic target. *J Clin Med.* (2021) 10:4791. 10.3390/jcm10204791 34682914 PMC8539594

[62] Kurutas EB. The importance of antioxidants which play the role in cellular response against oxidative/nitrosative stress: current state. *Nutr J.* (2016) 15:71. 10.1186/s12937-016-0186-5 27456681 PMC4960740

[63] Zhang YJ Gan RY Li S Zhou Y Li AN Xu DP Antioxidant phytochemicals for the prevention and treatment of chronic diseases. *Molecules.* (2015) 20:21138–56. 10.3390/molecules201219753 26633317 PMC6331972

[64] Atala E Fuentes J Wehrhahn MJ Speisky H. Quercetin and related flavonoids conserve their antioxidant properties despite undergoing chemical or enzymatic oxidation. *Food Chem.* (2017) 234:479–85. 10.1016/j.foodchem.2017.05.023 28551264

[65] Boots AW Drent M de Boer VC Bast A Haenen GR. Quercetin reduces markers of oxidative stress and inflammation in sarcoidosis. *Clin Nutr.* (2011) 30:506–12. 10.1016/j.clnu.2011.01.010 21324570

[66] Pizzino G Irrera N Cucinotta M Pallio G Mannino F Arcoraci V Oxidative stress: harms and benefits for human health. *Oxid Med Cell Longev.* (2017) 2017:8416763. 10.1155/2017/8416763 28819546 PMC5551541

[67] Koujan SE Gargari BP Mobasseri M Valizadeh H Asghari-Jafarabadi M. Effects of *Silybum marianum* (L.) Gaertn. (silymarin) extract supplementation on antioxidant status and hs-CRP in patients with type 2 diabetes mellitus: a randomized, triple-blind, placebo-controlled clinical trial. *Phytomedicine.* (2015) 22:290–6. 10.1016/j.phymed.2014.12.010 25765835

[68] Agarwal A Prasad R Jain A. Effect of green tea extract (catechins) in reducing oxidative stress seen in patients of pulmonary tuberculosis on DOTS Cat I regimen. *Phytomedicine.* (2010) 17:23–7. 10.1016/j.phymed.2009.10.019 19910173

[69] Torres EAFS Pinaffi-Langley ACDC Figueira MS Cordeiro KS Negrão LD Soares MJ Effects of the consumption of guarana on human health: a narrative review. *Compr Rev Food Sci Food Saf.* (2022) 21:272–95. 10.1111/1541-4337.12862 34755935

[70] Speisky H Shahidi F Costa AC Fuentes J. Revisiting the oxidation of flavonoids: loss, conservation or enhancement of their antioxidant properties. *Antioxidants (Basel).* (2022) 11:133. 10.3390/antiox11010133 35052636 PMC8772813

[71] Negut I Grumezescu V Grumezescu AM. Treatment strategies for infected wounds. *Molecules.* (2018) 23:2392. 10.3390/molecules23092392 30231567 PMC6225154

[72] Kadam S Nadkarni S Lele J Sakhalkar S Mokashi P Kaushik KS. Bioengineered platforms for chronic wound infection studies: how can we make them more human-relevant? *Front Bioeng Biotechnol.* (2019) 7:418. 10.3389/fbioe.2019.00418 31921821 PMC6923179

[73] Crompton R Williams H Ansell D Campbell L Holden K Cruickshank S Oestrogen promotes healing in a bacterial LPS model of delayed cutaneous wound repair. *Lab Invest.* (2016) 96:439–49. 10.1038/labinvest.2015.160 26855364

[74] Galandáková A Franková J Ambrožová N Habartová K Pivodová V Zálešák B Effects of silver nanoparticles on human dermal fibroblasts and epidermal keratinocytes. *Hum Exp Toxicol.* (2016) 35:946–57. 10.1177/0960327115611969 26500221

[75] Xie Y Yang W Tang F Chen X Ren L. Antibacterial activities of flavonoids: structure-activity relationship and mechanism. *Curr Med Chem.* (2015) 22:132–49. 10.2174/0929867321666140916113443 25245513

[76] Cushnie TP Lamb AJ. Antimicrobial activity of flavonoids. *Int J Antimicrob Agents.* (2005) 26:343–56. 10.1016/j.ijantimicag.2005.09.002 16323269 PMC7127073

[77] Sadgrove NJ Jones GL. From petri dish to patient: bioavailability estimation and mechanism of action for antimicrobial and immunomodulatory natural products. *Front Microbiol.* (2019) 10:2470. 10.3389/fmicb.2019.02470 31736910 PMC6834656

[78] Kumar S Pandey AK. Chemistry and biological activities of flavonoids: an overview. *ScientificWorldJournal.* (2013) 2013:162750. 10.1155/2013/162750 24470791 PMC3891543

[79] Kerdar T Rabienejad N Alikhani Y Moradkhani S Dastan D. Clinical, *in vitro* and phytochemical, studies of *Scrophularia* striata mouthwash on chronic periodontitis disease. *J Ethnopharmacol.* (2019) 239:111872. 10.1016/j.jep.2019.111872 30991136

[80] Amer SS Mamdouh W Nasr M ElShaer A Polycarpou E Abdel-Aziz RTA Quercetin loaded cosm-nutraceutical electrospun composite nanofibers for acne alleviation: preparation, characterization and experimental clinical appraisal. *Int J Pharm.* (2022) 612:121309. 10.1016/j.ijpharm.2021.121309 34801653

[81] Vikram A Jesudhasan PR Jayaprakasha GK Pillai SD Jayaraman A Patil BS. Citrus flavonoid represses *Salmonella* pathogenicity island 1 and motility in S. Typhimurium LT2. *Int J Food Microbiol.* (2011) 145:28–36. 10.1016/j.ijfoodmicro.2010.11.013 21168230

[82] Miklasińska-Majdanik M Kępa M Wojtyczka RD Idzik D Wąsik TJ. Phenolic compounds diminish antibiotic resistance of *Staphylococcus aureus* clinical strains. *Int J Environ Res Public Health.* (2018) 15:2321. 10.3390/ijerph15102321 30360435 PMC6211117

[83] Song L Hu X Ren X Liu J Liu X. Antibacterial modes of herbal flavonoids combat resistant bacteria. *Front Pharmacol.* (2022) 13:873374. 10.3389/fphar.2022.873374 35847042 PMC9278433

[84] Huang TW Lu HT Ho YC Lu KY Wang P Mi FL. A smart and active film with tunable drug release and color change abilities for detection and inhibition of bacterial growth. *Mater Sci Eng C Mater Biol Appl.* (2021) 118:111396. 10.1016/j.msec.2020.111396 33255001

[85] Woodburn KW Jaynes JM Clemens LE. Designed antimicrobial peptides against trauma-related cutaneous invasive fungal wound infections. *J Fungi (Basel).* (2020) 6:184. 10.3390/jof6030184 32971819 PMC7559897

[86] Netea MG Brown GD Kullberg BJ Gow NA. An integrated model of the recognition of *Candida albicans* by the innate immune system. *Nat Rev Microbiol.* (2008) 6:67–78. 10.1038/nrmicro1815 18079743

[87] Miura T Kawakami K Kanno E Tanno H Tada H Sato N Dectin-2-mediated signaling leads to delayed skin wound healing through enhanced neutrophilic inflammatory response and neutrophil extracellular trap formation. *J Invest Dermatol.* (2019) 139:702–11. 10.1016/j.jid.2018.10.015 30393083

[88] Bharti S Zakir F Mirza MA Aggarwal G. Antifungal biofilm strategies: a less explored area in wound management. *Curr Pharm Biotechnol.* (2022) 23:1497–513. 10.2174/1389201023666220411100214 35410595

[89] Jin YS. Recent advances in natural antifungal flavonoids and their derivatives. *Bioorg Med Chem Lett.* (2019) 29:126589. 10.1016/j.bmcl.2019.07.048 31427220

[90] Cheah HL Lim V Sandai D. Inhibitors of the glyoxylate cycle enzyme ICL1 in *Candida albicans* for potential use as antifungal agents. *PLoS One.* (2014) 9:95951. 10.1371/journal.pone.0095951 24781056 PMC4004578

[91] Navarro-Martínez MD García-Cánovas F Rodríguez-López JN. Tea polyphenol epigallocatechin-3-gallate inhibits ergosterol synthesis by disturbing folic acid metabolism in *Candida albicans*. *J Antimicrob Chemother.* (2006) 57:1083–92. 10.1093/jac/dkl124 16585130

[92] Américo ÁVLDS Nunes KM Assis FFV Dias SR Passos CTS Morini AC Efficacy of phytopharmaceuticals from the Amazonian plant *Libidibia ferrea* for wound healing in dogs. *Front Vet Sci.* (2020) 7:244. 10.3389/fvets.2020.00244 32656247 PMC7326013

[93] Tschumperlin DJ. Fibroblasts and the ground they walk on. *Physiology (Bethesda).* (2013) 28:380–90. 10.1152/physiol.00024.2013 24186933 PMC3858213

[94] Mascharak S desJardins-Park HE Davitt MF Griffin M Borrelli MR Moore AL Preventing engrailed-1 activation in fibroblasts yields wound regeneration without scarring. *Science.* (2021) 372:eaba2374. 10.1126/science.aba2374 33888614 PMC9008875

[95] Wang J Dodd C Shankowsky HA Scott PG Tredget EE. Deep dermal fibroblasts contribute to hypertrophic scarring. *Lab Invest.* (2008) 88:1278–90. 10.1038/labinvest.2008.101 18955978

[96] Rodrigues M Kosaric N Bonham CA Gurtner GC. Wound healing: a cellular perspective. *Physiol Rev.* (2019) 99:665–706. 10.1152/physrev.00067.2017 30475656 PMC6442927

[97] Liu J Zeng Y Sun G Yu S Xu Y He C Polygonum perfoliatum L., an excellent herbal medicine widely used in China: a review. *Front Pharmacol.* (2020) 11:581266. 10.3389/fphar.2020.581266 33304269 PMC7701256

[98] Fang CL Wang Y Tsai KH Chang HI. Liposome-encapsulated baicalein suppressed lipogenesis and extracellular matrix formation in Hs68 human dermal fibroblasts. *Front Pharmacol.* (2018) 9:155. 10.3389/fphar.2018.00155 29559910 PMC5845745

[99] Li X Zhai Y Xi B Ma W Zhang J Ma X Pinocembrin ameliorates skin fibrosis *via* inhibiting TGF-β1 signaling pathway. *Biomolecules.* (2021) 11:1240. 10.3390/biom11081240 34439906 PMC8393190

[100] Doersch KM Newell-Rogers MK. The impact of quercetin on wound healing relates to changes in αV and β1 integrin expression. *Exp Biol Med (Maywood).* (2017) 242:1424–31. 10.1177/1535370217712961 28549404 PMC5544166

[101] Pakyari M Farrokhi A Maharlooei MK Ghahary A. Critical role of transforming growth factor beta in different phases of wound healing. *Adv Wound Care (New Rochelle).* (2013) 2:215–24. 10.1089/wound.2012.0406 24527344 PMC3857353

[102] Lichtman MK Otero-Vinas M Falanga V. Transforming growth factor beta (TGF-β) isoforms in wound healing and fibrosis. *Wound Repair Regen.* (2016) 24:215–22. 10.1111/wrr.12398 26704519

[103] Xu X Zheng L Yuan Q Zhen G Crane JL Zhou X Transforming growth factor-β in stem cells and tissue homeostasis. *Bone Res.* (2018) 6:2. 10.1038/s41413-017-0005-4 29423331 PMC5802812

[104] Lin PS Chang HH Yeh CY Chang MC Chan CP Kuo HY Transforming growth factor beta 1 increases collagen content, and stimulates procollagen I and tissue inhibitor of metalloproteinase-1 production of dental pulp cells: role of MEK/ERK and activin receptor-like kinase-5/Smad signaling. *J Formos Med Assoc.* (2017) 116:351–8. 10.1016/j.jfma.2016.07.014 27720345

[105] Zhang Y Alexander PB Wang XF. TGF-β family signaling in the control of cell proliferation and survival. *Cold Spring Harb Perspect Biol.* (2017) 9:a022145. 10.1101/cshperspect.a022145 27920038 PMC5378054

[106] Li Z Yuan X Wang B Gao F. Icariin alleviates transforming growth factor-β1-induced epithelial-mesenchymal transition by targeting Smad and MAPK signaling pathways. *Am J Transl Res.* (2020) 12:343–60. 32194888 PMC7061835

[107] Singh R Akhtar N Haqqi TM. Green tea polyphenol epigallocatechin-3-gallate: inflammation and arthritis. [corrected]. *Life Sci.* (2010) 86:907–18. 10.1016/j.lfs.2010.04.013 20462508 PMC3146294

[108] Song Y Guo B Ma S Chang P Tao K. Naringin suppresses the growth and motility of hypertrophic scar fibroblasts by inhibiting the kinase activity of Akt. *Biomed Pharmacother.* (2018) 105:1291–8. 10.1016/j.biopha.2018.06.103 30021366

[109] Ud-Din S Wilgus TA McGeorge DD Bayat A. Pre-emptive priming of human skin improves cutaneous scarring and is superior to immediate and delayed topical anti-scarring treatment post-wounding: a double-blind randomised placebo-controlled clinical trial. *Pharmaceutics.* (2021) 13:510. 10.3390/pharmaceutics13040510 33917842 PMC8068279

[110] Chakraborty D Šumová B Mallano T Chen CW Distler A Bergmann C Activation of STAT3 integrates common profibrotic pathways to promote fibroblast activation and tissue fibrosis. *Nat Commun.* (2017) 8:1130. 10.1038/s41467-017-01236-6 29066712 PMC5654983

[111] Park G Yoon BS Moon JH Kim B Jun EK Oh S Green tea polyphenol epigallocatechin-3-gallate suppresses collagen production and proliferation in keloid fibroblasts *via* inhibition of the STAT3-signaling pathway. *J Invest Dermatol.* (2008) 128:2429–41. 10.1038/jid.2008.103 18463684

[112] Raktoe RS Rietveld MH Out-Luiting JJ Julio MK Zuijlen PP Doorn R Exon skipping of TGFβRI affects signalling and ECM expression in hypertrophic scar-derived fibroblasts. *Scars Burn Heal.* (2020) 6:2059513120908857. 10.1177/2059513120908857 32528734 PMC7263111

[113] Zhang YF Zhou SZ Cheng XY Yi B Shan SZ Wang J Baicalein attenuates hypertrophic scar formation *via* inhibition of the transforming growth factor-β/Smad2/3 signalling pathway. *Br J Dermatol.* (2016) 174:120–30. 10.1111/bjd.14108 26301336

[114] Zhang Y Wang J Zhou S Xie Z Wang C Gao Y Flavones hydroxylated at 5, 7, 3′ and 4′ ameliorate skin fibrosis *via* inhibiting activin receptor-like kinase 5 kinase activity. *Cell Death Dis.* (2019) 10:124. 10.1038/s41419-019-1333-7 30741930 PMC6370799

[115] Koivisto L Heino J Häkkinen L Larjava H. Integrins in wound healing. *Adv Wound Care (New Rochelle).* (2014) 3:762–83. 10.1089/wound.2013.0436 25493210 PMC4250945

[116] Januszyk M Kwon SH Wong VW Padmanabhan J Maan ZN Whittam AJ The role of focal adhesion kinase in keratinocyte fibrogenic gene expression. *Int J Mol Sci.* (2017) 18:1915. 10.3390/ijms18091915 28880199 PMC5618564

[117] Yeung V Sriram S Tran JA Guo X Hutcheon AEK Zieske JD FAK inhibition attenuates corneal fibroblast differentiation *in vitro*. *Biomolecules.* (2021) 11:1682. 10.3390/biom11111682 34827680 PMC8616004

[118] Meves A Geiger T Zanivan S DiGiovanni J Mann M Fässler R. Beta1 integrin cytoplasmic tyrosines promote skin tumorigenesis independent of their phosphorylation. *Proc Natl Acad Sci USA.* (2011) 108:15213–8. 10.1073/pnas.1105689108 21876123 PMC3174600

[119] Wang H Guo B Lin S Chang P Tao K. Apigenin inhibits growth and migration of fibroblasts by suppressing FAK signaling. *Aging (Albany NY).* (2019) 11:3668–78. 10.18632/aging.102006 31170089 PMC6594802

[120] Bolós V Gasent JM López-Tarruella S Grande E. The dual kinase complex FAK-Src as a promising therapeutic target in cancer. *Onco Targets Ther.* (2010) 3:83–97. 10.2147/ott.s6909 20616959 PMC2895777

[121] Wright B Watson KA McGuffin LJ Lovegrove JA Gibbins JM. GRID and docking analyses reveal a molecular basis for flavonoid inhibition of Src family kinase activity. *J Nutr Biochem.* (2015) 26:1156–65. 10.1016/j.jnutbio.2015.05.004 26140983

[122] Wang S Su R Nie S Sun M Zhang J Wu D Application of nanotechnology in improving bioavailability and bioactivity of diet-derived phytochemicals. *J Nutr Biochem.* (2014) 25:363–76. 10.1016/j.jnutbio.2013.10.002 24406273 PMC3959237

[123] Dadwal V Gupta M. Recent developments in citrus bioflavonoid encapsulation to reinforce controlled antioxidant delivery and generate therapeutic uses: review. *Crit Rev Food Sci Nutr.* (2021):1–21. 10.1080/10408398.2021.1961676 34378460

[124] Heleno SA Martins A Queiroz MJ Ferreira IC. Bioactivity of phenolic acids: metabolites versus parent compounds: a review. *Food Chem.* (2015) 173:501–13. 10.1016/j.foodchem.2014.10.057 25466052

[125] Başaran E Öztürk AA Şenel B Demirel M Sarica Ş. Quercetin, rutin and quercetin-rutin incorporated hydroxypropyl β-cyclodextrin inclusion complexes. *Eur J Pharm Sci.* (2022) 172:106153. 10.1016/j.ejps.2022.106153 35227839

[126] Hu B Liu X Zhang C Zeng X. Food macromolecule based nanodelivery systems for enhancing the bioavailability of polyphenols. *J Food Drug Anal.* (2017) 25:3–15. 10.1016/j.jfda.2016.11.004 28911541 PMC9333428

[127] Anand P Kunnumakkara AB Newman RA Aggarwal BB. Bioavailability of curcumin: problems and promises. *Mol Pharm.* (2007) 4:807–18. 10.1021/mp700113r 17999464

[128] Ban C Jo M Park YH Kim JH Han JY Lee KW Enhancing the oral bioavailability of curcumin using solid lipid nanoparticles. *Food Chem.* (2020) 302:125328. 10.1016/j.foodchem.2019.125328 31404868

[129] Bikiaris D Koutris E Karavas E. New aspects in sustained drug release formulations. *Recent Pat Drug Deliv Formul.* (2007) 1:201–13. 10.2174/187221107782331629 19075887

[130] Natarajan JV Nugraha C Ng XW Venkatraman S. Sustained-release from nanocarriers: a review. *J Control Release.* (2014) 193:122–38. 10.1016/j.jconrel.2014.05.029 24862321

[131] Baksi R Singh DP Borse SP Rana R Sharma V Nivsarkar M. *In vitro* and *in vivo* anticancer efficacy potential of quercetin loaded polymeric nanoparticles. *Biomed Pharmacother.* (2018) 106:1513–26. 10.1016/j.biopha.2018.07.106 30119227

[132] Bose S Du Y Takhistov P Michniak-Kohn B. Formulation optimization and topical delivery of quercetin from solid lipid based nanosystems. *Int J Pharm.* (2013) 441:56–66. 10.1016/j.ijpharm.2012.12.013 23262430

[133] Xie H Liu C Gao J Shi J Ni F Luo X Fabrication of zein-lecithin-EGCG complex nanoparticles: characterization, controlled release in simulated gastrointestinal digestion. *Food Chem.* (2021) 365:130542. 10.1016/j.foodchem.2021.130542 34265644

[134] Shishir MRI Gowd V Suo H Wang M Wang Q Chen F Advances in smart delivery of food bioactive compounds using stimuli-responsive carriers: responsive mechanism, contemporary challenges, and prospects. *Compr Rev Food Sci Food Saf.* (2021) 20:5449–88. 10.1111/1541-4337.12851 34668321

[135] Sadhukhan P Kundu M Chatterjee S Ghosh N Manna P Das J Targeted delivery of quercetin *via* pH-responsive zinc oxide nanoparticles for breast cancer therapy. *Mater Sci Eng C Mater Biol Appl.* (2019) 100:129–40. 10.1016/j.msec.2019.02.096 30948047

[136] Mu M Chen H Fan R Wang Y Tang X Mei L A tumor-specific ferric-coordinated epigallocatechin-3-gallate cascade nanoreactor for glioblastoma therapy. *J Adv Res.* (2021) 34:29–41. 10.1016/j.jare.2021.07.010 35024179 PMC8655135

[137] Ho CH Chu PY Peng SL Huang SC Lin YH. The development of hyaluronan/fucoidan-based nanoparticles as macrophages targeting an epigallocatechin-3-gallate delivery system. *Int J Mol Sci.* (2020) 21:6327. 10.3390/ijms21176327 32878305 PMC7504059

[138] Bose P Priyam A Kar R Pattanayak SP. Quercetin loaded folate targeted plasmonic silver nanoparticles for light activated chemo-photothermal therapy of DMBA induced breast cancer in Sprague Dawley rats. *RSC Adv.* (2020) 10:31961–78. 10.1039/d0ra05793b 35518142 PMC9056571

[139] Amini-Nik S Yousuf Y Jeschke MG. Scar management in burn injuries using drug delivery and molecular signaling: current treatments and future directions. *Adv Drug Deliv Rev.* (2018) 123:135–54. 10.1016/j.addr.2017.07.017 28757325 PMC5742037

[140] Mahmoud NN Qabooq H Alsotari S Tarawneh OA Aboalhaija NH Shraim S Quercetin-gold nanorods incorporated into nanofibers: development, optimization and cytotoxicity. *RSC Adv.* (2021) 11:19956–66. 10.1039/d1ra02004h 35479887 PMC9033756

[141] Vedakumari WS Ayaz N Karthick AS Senthil R Sastry TP. Quercetin impregnated chitosan-fibrin composite scaffolds as potential wound dressing materials - fabrication, characterization and *in vivo* analysis. *Eur J Pharm Sci.* (2017) 97:106–12. 10.1016/j.ejps.2016.11.012 27864063

[142] Chu J Shi P Yan W Fu J Yang Z He C PEGylated graphene oxide-mediated quercetin-modified collagen hybrid scaffold for enhancement of MSCs differentiation potential and diabetic wound healing. *Nanoscale.* (2018) 10:9547–60. 10.1039/c8nr02538j 29745944

[143] Croitoru AM Karaçelebi Y Saatcioglu E Altan E Ulag S Aydoğan HK Electrically triggered drug delivery from novel electrospun poly(lactic acid)/graphene oxide/quercetin fibrous scaffolds for wound dressing applications. *Pharmaceutics.* (2021) 13:957. 10.3390/pharmaceutics13070957 34201978 PMC8309188

[144] Prasathkumar M Sadhasivam S. Chitosan/Hyaluronic acid/Alginate and an assorted polymers loaded with honey, plant, and marine compounds for progressive wound healing-know-how. *Int J Biol Macromol.* (2021) 186:656–85. 10.1016/j.ijbiomac.2021.07.067 34271047

[145] Murray RZ West ZE Cowin AJ Farrugia BL. Development and use of biomaterials as wound healing therapies. *Burns Trauma.* (2019) 7:2. 10.1186/s41038-018-0139-7 30701184 PMC6346526

[146] Kalirajan C Palanisamy T. Bioengineered hybrid collagen scaffold tethered with silver-catechin nanocomposite modulates angiogenesis and TGF-β toward scarless healing in chronic deep second degree infected burns. *Adv Healthc Mater.* (2020) 9:e2000247. 10.1002/adhm.202000247 32378364

[147] Balestrin LA Kreutz T Fachel FNS Bidone J Gelsleichter NE Koester LS *Achyrocline satureioides* (Lam.) DC (Asteraceae) extract-loaded nanoemulsions as a promising topical wound healing delivery system: *in vitro* assessments in human keratinocytes (HaCaT) and HET-CAM irritant potential. *Pharmaceutics.* (2021) 13:1241. 10.3390/pharmaceutics13081241 34452202 PMC8400640

[148] Back PI Balestrin LA Fachel FNS Nemitz MC Falkembach M Soares G Hydrogels containing soybean isoflavone aglycones-rich fraction-loaded nanoemulsions for wound healing treatment - *in vitro* and *in vivo* studies. *Colloids Surf B Biointerfaces.* (2020) 196:111301. 10.1016/j.colsurfb.2020.111301 32871442

[149] Jee JP Pangeni R Jha SK Byun Y Park JW. Preparation and *in vivo* evaluation of a topical hydrogel system incorporating highly skin-permeable growth factors, quercetin, and oxygen carriers for enhanced diabetic wound-healing therapy. *Int J Nanomed.* (2019) 14:5449–75. 10.2147/IJN.S213883 31409998 PMC6647010

[150] Jin J Tang T Zhou H Hong XD Fan H Zhang XD Synergistic effects of quercetin-modified silicone gel sheet in scar treatment. *J Burn Care Res.* (2022) 43:445–52. 10.1093/jbcr/irab100 34089615

[151] Wu T Hou X Li J Ruan H Pei L Guo T Microneedle-mediated biomimetic cyclodextrin metal organic frameworks for active targeting and treatment of hypertrophic scars. *ACS Nano.* (2021) 15:20087–104. 10.1021/acsnano.1c07829 34792332

[152] Gan TJ. Poorly controlled postoperative pain: prevalence, consequences, and prevention. *J Pain Res.* (2017) 10:2287–98. 10.2147/JPR.S144066 29026331 PMC5626380

[153] Shin YC Yang WJ Lee JH Oh JW Kim TW Park JC PLGA nanofiber membranes loaded with epigallocatechin-3-O-gallate are beneficial to prevention of postsurgical adhesions. *Int J Nanomed.* (2014) 9:4067–78. 10.2147/IJN.S68197 25187710 PMC4149440

[154] Lee JH Shin YC Yang WJ Park JC Hyon SH Han DW. Epigallocatechin-3-O-gallate-loaded poly (lactic-co-glycolic acid) fibrous sheets as anti-adhesion barriers. *J Biomed Nanotechnol.* (2015) 11:1461–71. 10.1166/jbn.2015.2080 26295146

[155] Huang Y Shi R Gong M Zhang J Li W Song Q Icariin-loaded electrospun PCL/gelatin sub-microfiber mat for preventing epidural adhesions after laminectomy. *Int J Nanomedicine.* (2018) 13:4831–44. 10.2147/IJN.S169427 30214191 PMC6118333

[156] Pagadala NS Syed K Tuszynski J. Software for molecular docking: a review. *Biophys Rev.* (2017) 9:91–102. 10.1007/s12551-016-0247-1 28510083 PMC5425816

[157] Al-Shabibi MHS Al-Touby SSJ Hossain MA. Isolation, characterization and prediction of biologically active glycoside compounds quercetin-3-rutinoside from the fruits of *Ficus sycomorus*. *Carbohydr Res.* (2022) 511:108483. 10.1016/j.carres.2021.108483 34864403

[158] Ud-Din S Foden P Mazhari M Al-Habba S Baguneid M Bulfone-Paus S A double-blind, randomized trial shows the role of zonal priming and direct topical application of epigallocatechin-3-gallate in the modulation of cutaneous scarring in human skin. *J Invest Dermatol.* (2019) 139:1680–90.e16. 10.1016/j.jid.2019.01.030 30822414

